# Cardiovascular disease in racial/ethnic minority populations: illness burden and overview of community-based interventions

**DOI:** 10.1186/s40985-018-0109-4

**Published:** 2018-12-03

**Authors:** Brandon Muncan

**Affiliations:** State University of New York at Stony Brook, 100 Nicolls Rd. Stony Brook, New York, 11794 USA

**Keywords:** Cardiovascular disease, Racial/ethnic minorities, Race/ethnic identity, Community approach

## Abstract

Cardiovascular disease, the leading cause of death in the USA, poses a unique and multilateral burden to racial/ethnic minorities. The admixture of comorbid conditions, structural barriers, and psychosocial standing complicates the prevention, diagnosis, and management of cardiovascular disease in racial/ethnic minority populations and requires newer approaches to reduce existing disparities. A discussion of the cardiovascular disease risk burden is presented, along with an overview of multi-stratified considerations for improving racial/ethnic minority cardiovascular health via community engagement.

## Background

This paper presents a narrative literature review and commentary of racial/ethnic cardiovascular disease (CVD) disparities in the USA via a summary of a recent body of medical and public health research. Structural and psychosocial barriers to good cardiovascular health are presented, and community-based health initiatives and cultural competency measures are recommended to reduce health disparities.

## Defining racial/ethnic minorities

In the context of medico-sociological research, it is critical to produce working definitions of subpopulations and subgroups in order to classify data; however, defining race and ethnicity proves to be a particularly difficult issue. Extensive commentary exists on the ambiguity and lack of globally accepted definitions of race and ethnicity secondary to racialization, self-identification, and historical conflict [1]. In broad terms, *race* is perceived as the sum of outer characteristics including skin, hair, and eye color, and *ethnicity* is seen as a self-designated cultural identity related to national origin, language, religion, and tradition [2]. The larger sociological and medical literature is in agreement that race and ethnicity are social constructs with only very limited biological foundations, accounting for the difficulty in producing accepted terminology [3]. Given the constructed nature of race and ethnicity, it is historically true that classification based on a superiority hierarchy has led to institutional discrimination and racism [2].

Providing a universally accurate definition of “minority” proves to be just as difficult. In the context of this paper, a *minority* group is defined as any population that faces hardship, discrimination, or prejudice as a result of self-identification or extrinsically biased inclusion within an in-group. Furthermore, individual racial and ethnic groups are not discussed; rather, a collective term, “racial/ethnic minority,” is used.

## Literature review methodology

A review of published literature was conducted in order to identify relevant medical, public health, and sociological data. Retrieval of relevant research was accomplished via searching the electronic databases PubMed, MEDLINE, and Sociological Abstracts for the following keywords: *racial/ethnic discrimination*, *psychosocial* stress, *race/ethnic minority health*, *health disparities*, *cardiovascular disease*, and *community health*. Theoretical and empirical literature were considered. Criteria for inclusion included relevant discussion of cardiovascular and non-cardiovascular health disparities in racial/ethnic minorities, discussion of psychosocial factors in chronic disease etiology, and evaluation of community-based health interventions. A total of 154 articles and book chapters were generated, and review of the citations contained in the initial search generated an additional seven publications. Seventeen publications met the inclusion criteria and were analyzed in detail (see Table 1).

**Table 1:** Summary of reviewed publications

Year of publication	First author	Major findings
2014	Akdeniz et al.	Ethnic minority participants had a higher relative risk of schizophrenia compared to participants of a German lineage.
2016	Arnett et al.	African Americans are less likely to use primary care physicians than White counterparts; this is in part attributed to mistrust and discrimination.
2009	Brondolo et al.	Racism and discrimination contribute to increased psychosocial stress and unwellness.
2017	Carnethon et al.	Significant cardiovascular health disparities exist across US racial lines. Large-scale interdisciplinary interventions are recommended.
2016	Chen et al.	The Affordable Care Act in the USA has reduced gaps in access to care between racial/ethnic minority and majority patients.
2017	Fei et al.	Racial/ethnic minorities have disparate and higher rates of hypertension compared to White majority in New York City.
2015	Gallo et al.	Social and functional support within Hispanic participants was associated with lower diabetes mellitus prevalence.
2003	Garcia et al.	Racial/ethnic minority patients prefer language and race-concordant providers. Targeted interventions are recommended.
2015	Kershaw et al.	Individual- and neighborhood-level social stressors are associated with chronic heart disease
2007	Kurian et al.	Cardiovascular disease prevalence is disproportionately high in racial/ethnic minority groups. Tailored interventions are needed to bridge health care gaps.
2016	Liao et al.	Community-based interventions are successful in decreasing hypertension in Hispanic Communities within the USA
1999	Noh et al.	Racism and discrimination have been shown to increase risk of depression and adoption of unhealthy coping mechanisms.
2015	Record et al.	Community interventions and education reduced cardiac-cause mortality in Franklin County, Maine
2017	Snijder et al.	Racial/ethnic minority patients have higher rates of poor or uncontrolled diabetes compared to White counterparts.
2004	Stoddard et al.	Screening for cardiovascular risk factors in women during routine breast cancer exams was a successful strategy of identifying high-risk populations among underinsured and uninsured women.
2003	Troxel et al.	African American women reported higher social stress levels and had higher prevalence of carotid artery disease than White counterparts.
2017	Woringer et al.	Community-based interventions were successful in identifying high-risk cardiovascular disease populations and providing lifestyle education and timely treatment of illness.

## Identifying cardiovascular disease burden in racial-ethnic minority populations

CVD encompasses diagnoses including, but not limited to, coronary heart disease, stroke, sudden cardiac death, and peripheral vascular disease [4]. Well-documented risk factors for CVD include hypertension (high blood pressure), hypercholesterolemia (high cholesterol), diabetes, smoking, excessive alcohol consumption, and lack of physical activity. In the USA, CVD poses a significant concern from medical, policy, and public health standpoints [5, 3].

Although demography and biological/genetic determinants play an indisputable role in predisposing certain minority populations to CVD risk factors and consequent cardiovascular pathology, increasingly, data have suggested a causal role of social circumstances in disease progression [4]. The burden of CVD in disadvantaged populations is a partial consequence of lower socioeconomic status (SES) and social distress, both of which are recognized fundamental causes of morbidity and mortality [6]. Mechanisms such as structural barriers to health care and psychosocial stress have been theorized to contribute to unequal disease rates; in particular, racial/ethnic minorities have experienced a disproportionate incidence and prevalence of CVD [7, 3].

The literature has illustrated that hypertension, a critical CVD risk factor, is more prevalent in Hispanic and American Indian minorities, compared to non-Hispanic Whites in the USA [8]. In New York City, Southeast Asians and non-Hispanic Blacks had significantly higher mean blood pressures than non-Hispanic Whites [9]. In a similar capacity, the Healthy Life in an Urban Setting (HELIUS) study found that compared to Whites, Black, and Hispanic individuals had higher rates of diabetes mellitus and associated complications [10]. Additionally, largely modifiable social risk factors for CVD, namely smoking, excessive alcohol consumption, and limited physical activity, are present in significantly more American Indians, Hispanics, and Blacks than in White counterparts [8]. Differences in race/ethnicity prevalence of hypercholesterolemia, another paramount CVD risk factor, have not been conclusively quantified [8]. In all, many sociological theorists have suggested explanatory schemes of such health disparities; hypothesized mechanisms for these findings include limited health literacy and poor lifestyle choices, elements which are discussed further in this paper.

## Structural barriers to adequate cardiovascular health

Several hypotheses have been proposed to explain how and why racial/ethnicity health gaps exist. Some of these include the stratified effects of SES on access to health care and similar health resources, while others focus on policy-level circumstances that put racial/ethnic minorities at an institutional disadvantage. Low SES has been correlated with low income, low educational achievement, poorer living conditions, and inability to access primary or specialty care due to insurance or other cost problems [7, 3, 11]. Clearly, these can pose barriers to recognizing symptoms of CVD, seeking medical care, and affording safer, cleaner living conditions, and food of greater nutritional value.

**Fig. 1:**
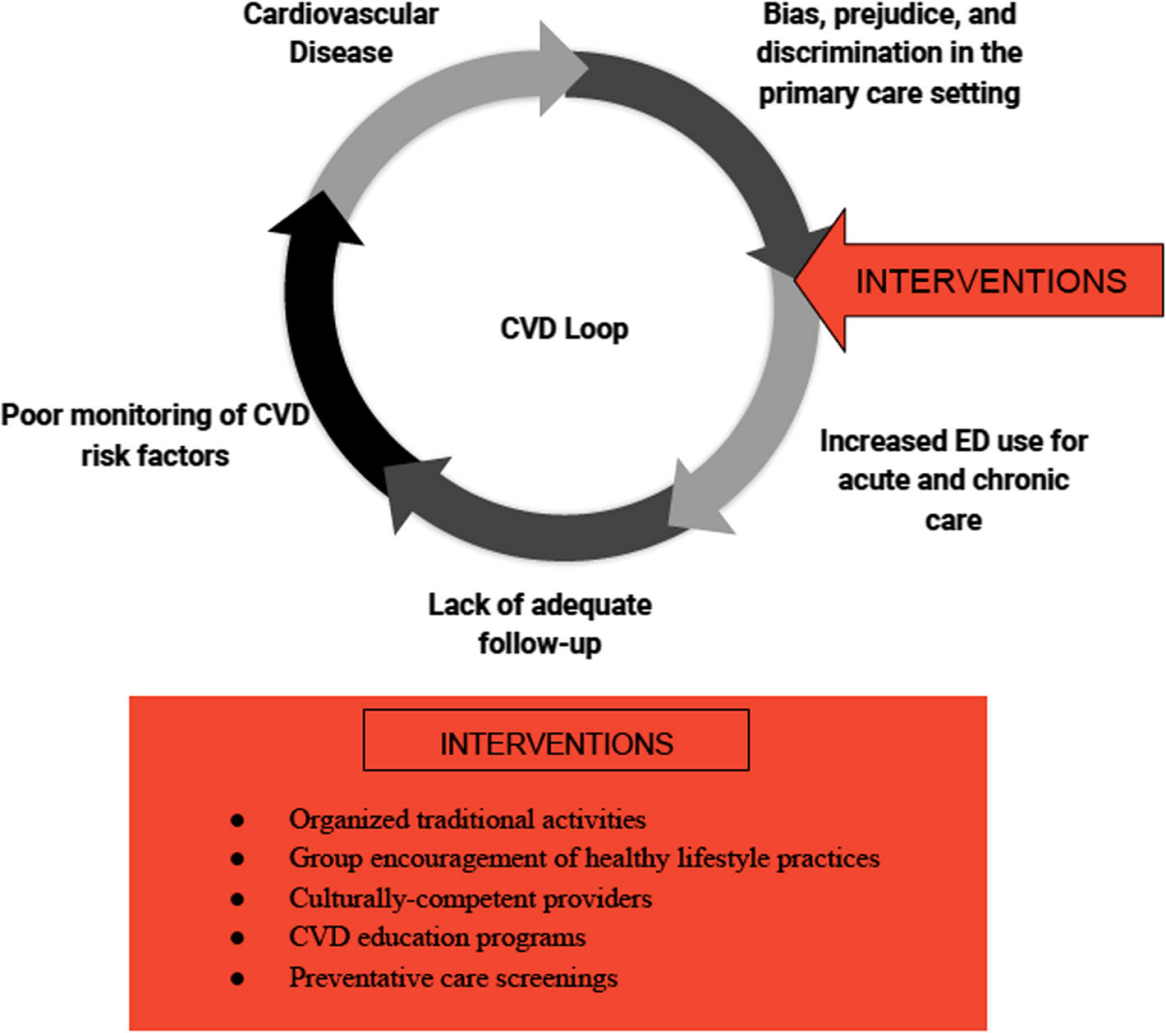
Components of the CVD loop in racial/ethnic minority populations and possible community-based interventions

Arnett et al. [12] consider geographical location of primary care physicians as a contributor to health differences because many providers tend to stray away from under-insured and racially segregated communities. This leads many people in such minority neighborhoods to travel farther away from home in scope of primary care. More often than not, people turn to emergency departments (EDs) for acute and chronic health management, which poses both patient care and economic burden [12]. African Americans, for example, have been shown to use ED care at disproportionately higher rates than Whites and only see a primary care provider at a fraction of the rate of their White counterparts; bias, prejudice, and discrimination dissuade minority visits to primary care providers for fear of inadequate treatment and instigate a perilous loop of substandard medical attention that further includes increased ED use and consequent lack of care continuity (see Fig. 1). This loop increases the burden of CVD risk factors and promotes the unchecked development of CVD in minority populations.

Alongside the limited access to primary and cardiovascular specialty care, insurance is another structural variable that has heavily influenced race/ethnicity health disparities in the USA. Since 2014, improvements have been made to bridge the care gap between patients of different SES and race/ethnicity through the Affordable Care Act [13]. Under the Patient Protection and Affordable Care Act (ACA), a national legislative effort to ensure coverage for all Americans and protect against insurer bias, it is estimated that approximately 16.9 million previously uninsured people have been able to access care, either through Medicaid expansion or through other means [13, 14]. Medicaid, a national health insurance program that provides medical benefits and coverage for low-income children and adults, underwent comprehensive expansion of coverage under the ACA to include many previously uninsured low- and middle-income persons. Although ACA has made significant public health contributions by improving rates of racial/ethnic minority insurance enrollment, disparities still exist. Many state legislatures in conservative-leaning US states opted to decline such coverage expansion for economic and political reasons, limiting coverage for lower SES patients in these states [13]. In states without Medicaid expansion, particularly in politically conservative southern US states, low-income persons and families who do not meet Medicaid criteria but cannot afford private health insurances remain uninsured. Moreover, the federal tax penalties encountered for not having health insurance add to their economic burden [2, 15]. Thus, from an economic standpoint, insurance-based access to care is an important factor and even more so in the changing political climate of the USA.

## Psychosocial mechanisms

Psychological and social effects also play crucial roles in predisposing minority populations to stress and to consequent pathology including CVD [2, 3, 16]. Crimes like robbery and firearm violence, for example, are subjects of many studies and have been shown to be commonly associated with low SES neighborhoods, including those of primarily racial/ethnic minority populations [17]. Furthermore, Roberts et al. [18] concluded that posttraumatic stress disorder (PTSD) secondary to violence or traumatic exposure occurs at higher rates in Blacks as compared to Whites and Hispanics in the USA. Alongside devastating psychological and social ramifications of PTSD, Turner et al. [19] concluded that PTSD is an independent risk factor for cardiovascular consequences, namely myocardial ischemia. Moreover, PTSD and comorbidities are frequently undiagnosed and untreated in racial-ethnic minorities [18]. These social conditions and their effects, in part, contribute to CVD pathogenesis in racial/ethnic minorities.

Psychosocial stress secondary to discrimination and bias within racial/ethnic minority communities and within the greater health care system also cause disparities through various mechanisms. From a cultural standpoint overall, differences in customs have been established as impediments to successful patient-provider communication, leaving racial/ethnic minority individuals uninformed about their cardiovascular health and dissatisfied with their care. [11]. Interpretations of this lack of cultural competency frequently include perceived explicit or implicit bias, which in turn reinforces the CVD loop (Fig. 1) and promotes poor cardiovascular health. Fear of disadvantageous racist treatment is often pervasive among racial/ethnic minorities, leading to reluctance to seek primary or cardiovascular care [2, 12]. As a consequence of historical and current remnants of institutional prejudice, mistrust in providers of different race/ethnicities than patients becomes more common and prevents adequate diagnosis and medical monitoring. There is a consequent lack of follow-up care, which further contributes to the CVD loop. Studies have concluded empirically that many minority patients preferred race/ethnicity-concordant providers in order to improve quality of care and comfort levels [20]. Furthermore, Garcia et al. [20] reviewed literature that suggested improved self-rated health in Black and Hispanic patients with race-concordant physicians as a result of higher quality interpersonal interactions.

Language barriers also pose difficulties in efficient communication and provision of cardiovascular health care. Minority patients with limited or no English language ability may be at a critical disadvantage in the medical setting as relay of information through interpreters (if and when available) can be incomplete or misdirected [20, 21]. Providers as a result cannot obtain accurate patient history or symptom descriptions, and patients cannot fully understand recommendations and treatment options. In the case of CVD prevention and mediation, such language barriers pose a unique problem: many of the modifiable CVD risk factors can be managed with solid treatment-adherence measures (medication compliance, diet regimens, exercise plans, social groups for smoking and binge-drinking cessation) and close follow-up care, which are difficult to perform and control in the setting of communicative disability or inability, and contribute significantly to the inadequate care continuity component of the CVD loop. Patients may feel under-cared for and are likely to encounter unfamiliarity with offered therapies or the need to schedule regular specialty appointments, thereby compounding stress and promoting disease. New focuses on improving effective patient-provider dialog are required in order to bridge this contributing gap to race/ethnicity health inequalities.

## Racial/ethnic identity and social support structures

Racial/ethnic identity is a broad social continuum that involves an individual or group connection with a particular race/ethnicity. It encompasses knowledge and practice of traditional customs and pride in such association. Identification occurs on a spectrum, with some individuals or groups claiming no belonging, while others claim that identifying with a particular race-ethnicity is central to their lives [22, 23]. Empirical evidence of the stress-buffering effects of identifying and belonging to a particular racial/ethnic group is mixed. Some authors have found that race/ethnicity increases the burden of bias and consequent negative health effects, while many others have found quite the opposite: that racial/ethnic identity may be protective against extrinsic discrimination via lowering stressful responses to such prejudice and improving coping capability [22, 24]. Possible explanations for the heterogeneity of these results are the differing degrees of discrimination faced by sample populations, the strength of the identity described, and the presence of co-identities or acculturation factors. If an individual identifies strongly with a minority group, but feels displaced within the greater society, the stress-buffering effects of racial/ethnic identity may be offset.

For the most part, nevertheless, the literature has identified positive stress-buffering effects of strong racial/ethnic identification. Mossakowski [22] found that Filipino-Americans with a higher degree of ethnic identity reported fewer depressive symptoms and a stronger coping ability in the face of discrimination. Furthermore, Mossakowski concluded that strong ethnic identity did indeed buffer stress effects of a prejudicial society and called for further research to determine whether this trend is true in other ethnic minority populations as well. Similarly, Earnshaw et al. [23] reported an association of stigma experience in HIV patients (also considered a minority population, albeit not a race-ethnicity minority) with the presence of physical symptoms secondary to stress mechanisms. The authors suggested that social and community support resources played a pivotal role in reducing anticipated stigma and improving HIV symptom reporting. It is important to note, however, that these effects are only part of the greater issue and that consideration must be given to the interaction of multiple stress-producing discriminatory events in order to obtain a complete understanding of the problem [25].

To date, only a handful of large-scale studies have tested the direct impact of discrimination on the development of CVD. Kershaw et al. [26] studied such relationships through neighborhood stress reports; community-wide increases in CVD risk within highly stressed urban cohorts of six large American cities were identified. Furthermore, Troxel et al. [27] concluded that Black women who reported past racist experiences had small-scale increases in subclinical CVD (atherosclerosis), compared to White women who did not report such bias. Overall, there is a demonstrated correlation between discrimination and CVD; however, there is additional need for experimental evidence with greater sample sizes in order to investigate and validate an independent causal relationship between race/ethnicity stress and cardiovascular health consequences.

## Community-based approaches to improving cardiovascular health

To date, the largest multiracial-ethnic community-based health initiative in the USA is the Racial and Ethnic Approaches to Community Health Across the United States (REACH US). REACH US employed tailored neighborhood coalitions and community health outreach programs across 40 communities in the USA in order to educate local minority groups about health and wellness, encourage healthy lifestyle practices, promote health screenings, and encourage policy changes that would decrease health disparities via local community health workers who had strong ties to the neighborhood and its residents [28]. In participant Hispanic neighborhoods, educational and community support efforts increased awareness of hypertension (CVD risk factor) and the importance of blood pressure-lowering measures such as medication adherence and dietary choices. Liao et al. [28] concluded that such tailored community approaches, including neighborhood food watch programs and church-based lifestyle education, are promising strategies to mediate health disparities such as disproportionate prevalence of hypertension in minorities.

Similarly, the presence of increased structural and functional support networks in Hispanic neighborhoods researched as part of the Hispanic Community Health Study/Study of Latinos (HCHS/SOL) have been correlated with lower diabetes (CVD risk factor) prevalence [29]. The researchers identified (a) an existing role of social interaction in stress reduction and (b) positive coping mechanisms as plausible vehicles for the reported lower diabetes risk; nevertheless, Gallo et al. [29] calls for further studies in minority-population diabetes causal mechanisms. A particular focal point of future investigations should include specific, direct relationships between such risks in minority populations and CVD diagnoses.

An illustrative example of community-strategy efficacy in the experimental setting of cardiovascular health is a 40-year observational study in Franklin County, Maine, a low SES county, which highlighted important trends in CVD risk factor prevalence and incidence as community risk-reduction initiatives were introduced [30]. Control of hypertension and hypercholesterolemia increased by 24.7% and 28.5%, respectively, and smoking cessation rates increased by 17.4% as a result of door-to-door outreach efforts involving health coaching and rigorous monitoring and follow-up care [30]. In addition, hospitalizations and cardiovascular-cause mortality were reduced in Franklin County compared to rates in other Maine counties. These data point to the promise of community-based preventative, educational, and CVD monitoring programs in decreasing poor health outcomes.

Two additional seminal examples of successful cardiovascular health improvement initiatives via community-based approaches are The Georgia Stroke and Heart Attack Prevention Program (SHAPP) and the WISEWOMAN project. In SHAPP, 15,000 community health nurses and physicians offered low SES hypertensive patients counseling and case management in public clinics, in regard to pharmacotherapy and lifestyle modifications. Educational initiatives as well as low- or no-cost blood pressure medications were provided to qualified low-income participants. In 2003, hypertension burden in SHAPP communities was 60% compared to non-SHAPP communities which showed a 68% prevalence. Moreover, a 46% reduction in cardiac complications was reported among SHAPP patients compared to controls [31, 32]. From a fiscal standpoint, community-based intervention produced more cost-efficient blood pressure control (486 USD per annum) than usual care nationally (624 USD per annum) [31].

In the national WISEWOMAN project, CVD risk screening was performed during routine breast and cervical cancer screenings in underinsured and uninsured women over 50. In relation to the psychosocial mechanisms of CVD and the CVD loop, intervening during routine exams at local offices increases provider accessibility. In the Massachusetts division of WISEWOMAN, CVD risk identification and lifestyle improvement through exercise and healthy diet encouragement led to 7–9% hypertension reduction in participants, indicating a successful community-based approach [31, 32].

## Future considerations for community-based cardiovascular health improvements

In this paper, the proposed approaches to bridging racial/ethnic health disparities integrate evidence-based data and psychosocial theory, while taking into account recommendations from large sociological and medical studies [33, 3, 28]. A multi-layered system solution is recommended based on conclusive data showing that CVD risk factor and CVD morbidity prevalence has several contributing factors including lack of access to care, poor health education, weak community infrastructure, and concerns of racial/ethnic identity. The goal of such solutions is to decrease the negative effects of bias and discrimination by intervening early in the CVD loop so as to mitigate further consequences. Along the same lines, community-based educational and service interventions (which also involve racial/ethnic inclusivity) play a role in encouraging screening and therapy adherence, thereby limiting excessive ED use and promoting rigorous continuity of care (see Fig. 1). Considerations must also be made to address acculturation levels as well as cultural competency in the greater society, which may positively impact interpersonal interactions between minorities and the race-ethnic majority. A two-tiered system is discussed, comprising of community health centers and minority cultural involvement initiatives.

Community health centers (CHCs) have been shown to improve access to health care in racial/ethnic minority neighborhoods [22, 26, 28, 30]. It is hypothesized that an increase in primary preventative and specialty cardiovascular care within existing and new CHCs may positively impact CVD morbidity and mortality. It may be more economically feasible to seek care in local communities rather than to travel longer distances to other providers or EDs. Seeking local care at CHCs may improve health care spending schemes and decrease last-resort and inappropriate ED visits as well [12]. Cultural competency within these centers is vital to patient satisfaction levels, effective communication with providers, and rates of compliance in racial/ethnic minorities. To improve cultural competency, CHCs should offer accurate language interpretation services to patients and recruit employees and volunteers that speak languages frequently encountered in the local community. Efforts should be made to adhere to a patient’s provider language and gender preferences whenever possible. Cardiovascular specialty care should be available in CHCs; where this is not feasible, robust referral networks to culturally competent specialist providers should be in place. Bagget et al. describe the importance of specialty cardiovascular diagnostic technology in homeless populations, who experience similar psychosocial disadvantages to racial/ethnic minority patients in terms of health. As a result, diagnostic tests such as electrocardiography and echocardiography, wherever possible, should be available on-site in CHCs to promote patient convenience and improve compliance rates [34]. With respect to pharmacotherapy, once-daily medications have been shown to improve the likelihood of patient compliance and are recommended [34]. Ideally, CHCs should also provide health insurance workshops and aid-office referral network information in order to educate and improve health care finances and insurance enrollment, irrespective of inclusive policies of the ACA. Lastly, health education should be central to CHC efforts to improve minority health, with an emphasis on improving awareness of early CVD symptoms, encouraging preventative screenings for CVD risk factors, and adopting healthy lifestyle practices. As with the WISEWOMAN project, effective strategies for widespread information dissemination and screenings include interventions at local offices, schools, and other areas of high community engagement [32].

Minority cultural involvement initiatives should also be considered in order to improve race-ethnic identity as well as to promote healthy community relationships and ties to racial/ethnic majority populations. Attaining these goals may then lead to improved psychosocial wellness and decreased rates of CVD. Heretofore, no empirical studies have been conducted to assess or identify the strength of such initiatives on majority-minority and minority in-group interactions and relationships; nevertheless, from the theoretical literature, the following hypotheses are made [35, 36] within focal areas of neighborhood involvement and gathering (such as churches and other religious organizations and community centers): organized and well-advertised community activities involving traditions and customs of local racial/ethnic minority heritages may both increase benefits of in-group race/ethnicity identity and educate out-group individuals via improved inter-ethnic communication, thereby developing cultural competency. In turn, these changes may improve perceived and experienced stress levels and mediate negative effects on cardiovascular health. In addition, exposing the community to cultural diversity may increase acceptance of race-ethnicity differences and limit bias on a structural level. Moreover, inclusionary policies on the local level and beyond (in terms of bias/discrimination laws, equal opportunity employment and housing, etc.) are recommended in order to promote the closing of structural race/ethnicity gaps and increase availability and access to cardiovascular care.

## Conclusions

Racial/ethnic disparities in cardiovascular health have been well documented in the literature [3, 8, 9, 26–28]. Structural and psychosocial obstacles including insurance burden, limited access to care, lack of provider cultural competency, health illiteracy, English language barriers, and stress secondary to discrimination contribute to an increase in CVD risk factors and consequent CVD. Furthermore, identification as a racial-ethnic minority poses a unique difficulty in the proper management of diagnosed CVD and comorbidities for the same reasons and leads to greater cardiovascular-cause mortality, compared to White counterparts [3]. A two-tiered system, involving CVD focus in community health centers as well as minority community involvement to improve ethnic identity and inter-ethnic relationships, is proposed to mediate racial/ethnic difference in cardiovascular health and produce a more equitable society by interrupting the CVD loop at a critical point of care.

## Abbreviations

ACA: Affordable Care Act
CHC: Community health center
CVD: Cardiovascular disease
ED: Emergency department
PTSD: Posttraumatic stress disorder
SES: Socioeconomic status
USD: United States Dollar
